# Confounding of linkage disequilibrium patterns in large scale DNA based gene-gene interaction studies

**DOI:** 10.1186/s13040-019-0199-7

**Published:** 2019-06-10

**Authors:** Marc Joiret, Jestinah M. Mahachie John, Elena S. Gusareva, Kristel Van Steen

**Affiliations:** 1 BIO3, GIGA-R Medical Genomics, Avenue de l’Hôpital 1-B34-CHU, Liège, 4000 Belgium; 2 Biomechanics Research Unit, GIGA-R in-silico medicine, Liège, Avenue de l’Hôpital 1-B34-CHU, Liège, 4000 Belgium; 3 WELBIO researcher, Avenue de l’Hôpital 1-B34-CHU, Liège, 4000 Belgium

**Keywords:** Genome-wide association interaction studies (GWAIS), Model-based multifactor-dimensionality reduction (MB-MDR), Gametic phase disequilibrium (GPD), Signal sensitivity, 1000 genomes project, Ankylosing spondylitis

## Abstract

**Background:**

In Genome-Wide Association Studies (GWAS), the concept of linkage disequilibrium is important as it allows identifying genetic markers that tag the actual causal variants. In Genome-Wide Association Interaction Studies (GWAIS), similar principles hold for pairs of causal variants. However, Linkage Disequilibrium (LD) may also interfere with the detection of genuine epistasis signals in that there may be complete confounding between Gametic Phase Disequilibrium (GPD) and interaction. GPD may involve unlinked genetic markers, even residing on different chromosomes. Often GPD is eliminated in GWAIS, via feature selection schemes or so-called pruning algorithms, to obtain unconfounded epistasis results. However, little is known about the optimal degree of GPD/LD-pruning that gives a balance between false positive control and sufficient power of epistasis detection statistics. Here, we focus on Model-Based Multifactor Dimensionality Reduction as one large-scale epistasis detection tool. Its performance has been thoroughly investigated in terms of false positive control and power, under a variety of scenarios involving different trait types and study designs, as well as error-free and noisy data, but never with respect to multicollinear SNPs.

**Results:**

Using real-life human LD patterns from a homogeneous subpopulation of British ancestry, we investigated the impact of LD-pruning on the statistical sensitivity of MB-MDR. We considered three different non-fully penetrant epistasis models with varying effect sizes. There is a clear advantage in pre-analysis pruning using sliding windows at *r*^2^ of 0.75 or lower, but using a threshold of 0.20 has a detrimental effect on the power to detect a functional interactive SNP pair (power < 25*%*). Signal sensitivity, directly using LD-block information to determine whether an epistasis signal is present or not, benefits from LD-pruning as well (average power across scenarios: 87%), but is largely hampered by functional loci residing at the boundaries of an LD-block.

**Conclusions:**

Our results confirm that LD patterns and the position of causal variants in LD blocks do have an impact on epistasis detection, and that pruning strategies and LD-blocks definitions combined need careful attention, if we wish to maximize the power of large-scale epistasis screenings.

## Introduction

A single-nucleotide polymorphism (SNP) is a variation in a single nucleotide that occurs at a specific position in the genome, where each variation is commonly present within a population (e.g. > 1 *%*). Their frequency and wide-spread distribution across the genome make them interesting markers for known complex diseases in genome-wide association studies (GWAS). The success of GWAS using SNPs as genetic markers in part relies on Linkage Disequilibrium (LD) as a population concept. LD is a property of SNPs in a genomic sequence that refers to allelic association and linkage. It can be considered to be linkage between markers on a population scale [1]. It is different from Gametic Phase Disequilibrium (GPD) that describes the non-random association of alleles within gametes (even for physically unlinked loci on different chromosomes). LD is a special case of GPD when the loci are linked.

Gametic Phase Disequilibrium-Linkage Disequilibrium in natural populations may result from different evolutionary forces, including random genetic drift due to sampling of gametes during reproduction, but also epistatic selection [2]. This explains why researchers on two-loci epistatic selection with recombinant inbred lines have used the idea to screen for distortions of pairwise segregation [3] or to look for epistasis selection networks via first screening for loci that show significant long-range LD [4]. For unrelated humans, long-range haplotypes have been shown to extend to over a few hundred kilobases [5], yet altogether only span a very small fraction of an entire chromosome. Hence when evidence is found for substantial long-range LD, special forces should be thought of (see for instance [6]). One of these forces may be epistatic selection, which can maintain LD indefinitely [7], but may also be selection with strictly additive genes [8]. The presence of different GPD structures between cases and controls has been explicitly used in epistasis detection tools such as EPIBLASTER [9]. This exploitation should be made with care as complete confounding may exist between GPD and interaction [10].

GPD/LD is important to GWAS as it allows identifying genetic markers that tag the actual causal variants to complex human diseases. In the search for causal variants, several authors have speculated that understanding the interplay between genetic loci may further contribute to understanding disease-underlying mechanisms [1, 11–13]. Epistasis, in its broadest sense, refers to the dependence of the outcome of a mutation on the genetic background (refer to [14] and [12, 15] for reviews). From a biological perspective, genetical epistasis refers to a masking effect whereby a variant or allele at one locus masks the expression of a phenotype at another locus [16]. Statistical epistasis describes the situation where the combined effect of two or more loci cannot be predicted from the sum of their individual single-locus effect in a mathematical model [17]. The discovery of biological epistasis via statistical methods is a big challenge, especially in the absence of prior hypotheses [1, 18, 19] and limits coupling biological relevance to statistical findings. The interpretation and reproducibility of findings is hampered by the vast number of epistasis data mining tools that exist, non-consensus about GWAI protocols to carry out the analyses on noisy or confounded data, and the fact that signals are detected on tagSNP pairs rather than functional or causal SNPs [20, 21]. Recent advances in simulating synthetic data that faithfully enough represent the complexity of the biological nature of human disease systems will be helpful in this sense [22].

In the scenario of so-called genome-wide association interaction studies (GWAIS), GPD/LD can be a merit, similar to GWAS, but it may also be a burden. GPD/LD may actually interfere with the detection of genuine epistasis signals in that there may be complete confounding between Gametic Phase Disequilibrium (GPD) and interaction [10]. One of the strategies to deal with such confounding is to eliminate GPD. This can be done via SNP selection to only keep a set of SNPs that are mutually uncorrelated (e.g., by taking *r*^2^ as a measure of allelic association and a threshold of *r*^2^=0.20). Several algorithms for pruning SNPs in this way or for reducing the degree of LD between SNPs exist, often involving a sliding windows approach to reduce the number of SNP pairs to interrogate. Popular pruning strategies are implemented in PLINK 1.07/1.9 [23, 24], which sequentially scan the genome for pairs of correlated SNPs, not using phase information but only using allele counts. In contrast to pruning, clumping retains a single representative SNP per highly-correlated region of SNPs. With ever increasing datasets generated via the latest sequencing technologies, the search for computational efficient algorithms is an ongoing effort (for instance, SNPrune [25]).

In this study, we investigate the impact of correlated SNPs on the performance of large-scale epistasis screening and argue about correlation thresholds that keep a balance between maintaining sufficient epistasis screening power and reducing the occurrence of redundant epistasis. In addition, we point towards the necessity of exploiting LD-block information while interpreting epistasis results and give recommendations about unbiased LD estimation in this context. As tool analytic example, we take Model-Based Multifactor-Dimensionality Reduction (MB-MDR). MB-MDR is a non-parametric method, in the sense that no assumptions are made regarding genetic modes of (epistatic) inheritance. It can be model-based (MB) when a particular model is chosen to separate main SNP effects from pure epistasis in joint locus signals. The data reduction part in MB-MDR relies on association tests, which may or may not be parametric [26].

## Methods

A total of 1200 synthetic datasets were built, corresponding to 4 scenarios of ‘causal’ Disease Susceptibility Loci (DSL) pairs, embedded in real human LD blocks extracted from a single HapMap3 subpopulation of British ancestry, × 3 effect sizes for a pure epistatic interaction × 100 retrospective case-control replicates with 1000 subjects in each cohort. We explain below how these synthetic datasets were constructed and which analysis workflows were conducted.

### *Forward time simulation models to generate realistic genetic profiles for individuals*

Here, we evolved a founder population of all 91 subjects from the GBR subpopulation of the HapMap3/1000 Genomes Project (GRCh37.p13 assembly) [27–30], using simuPOP 1.1.8.3 [31, 32]. The aim was to generate 100 synthetic datasets with 1000 cases and 1000 controls, exhibiting realistic complex LD patterns and haplotype blocks, extracted from a selected population of size 10,000 expanded from the homogeneous British ancestry subpopulation of 91 subjects (England and Scotland), for different disease model settings. This GBR homogeneous subpopulation of 91 subjects was chosen for two reasons:

1. to guard against large-scale population substructure or stratification issues;
2. to facilitate making links between synthetic data analysis and real-life data analysis on Ankylosing Spondylitis dataset from WTCCC2, which mainly involved individuals of British ancestry [33].

Forward-time simulation (Peng [31, 32]) was practically carried out with Python scripts from the simuPOP simulation environment developed by Peng et al. [31], in 4 steps, which are described in more detail below.

#### Step 1

Two segments of two chromosomes from the 91 individuals of the GBR subpopulation of HapMap3 [29, 30] were selected with their starting and ending physical positions on the human genome:

- (chr 7:110,200,000-110,450,000) which spans a 250 kbp region with 964 markers (SNPs) at an average marker distance of 260 bp.
- (chr 8:91,525,000-91,775,000) which spans a 250 kbp region with 787 markers (SNPs) at an average marker distance of 318 bp.

**Fig. 1:**
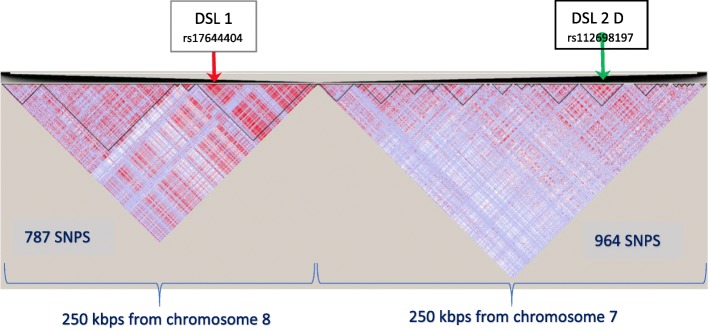
Two LD block structures on two chromosomes: Presented are two LD blocks corresponding to HapMap3 GBR subpopulation of 91 unrelated individuals. The selected regions are from chromosome 8 (left) and 7 (right) consist of 787 and 964 SNPs respectively. The positions of causal epistatic variants are indicated by arrows

**Fig. 2:**
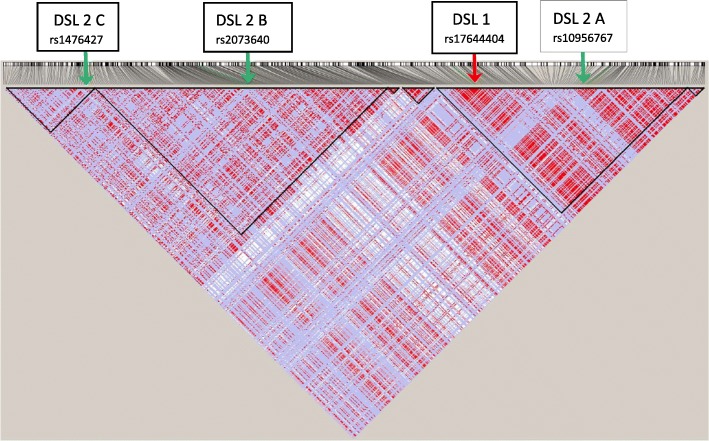
LD block structures on chromosome 8 selected region. Zoom-in on the positions of causal DSLs on chromosome 8 corresponding to 3 out of 4 epistatic scenarios

We removed SNP rs28568272 on chr8 at locus position 91,652,958 so as to retain only bi-allelic markers for convenience, even though MB-MDR is able to analyse any categorical variable. All SNPs were subjected to the following QC checks: minor allele frequency MAF higher than 1%, missingness rate less than 10%, Hardy-Weinberg Equilibrium (significance level at 5·10^−15^). This resulted in a total of 1751 bi-allelic markers, typed for all the 91 individuals in the *founder population* of British ancestry. The LD pattern corresponding to the two juxtaposed DNA segments is displayed on Figs. 1 and 2. It shows interesting features of separate LD blocks of different sizes and LD intensities. DSL 1 and DSL 2(A-D) refer to disease susceptibility loci pairs (DSL 1, DSL 2 A-D) and were chosen in such a way that they exhibit different properties regarding their location in the LD blocks.

#### Step 2

**Fig. 3:**
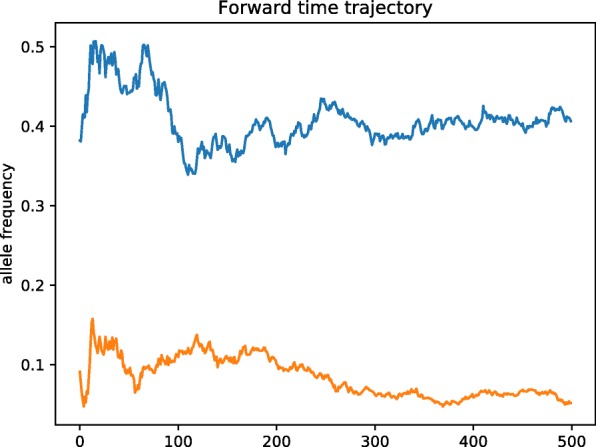
Trajectory of the minor allele frequencies. Simulated forward-time trajectory of allele frequency over 500 generations. The **blue** line is the trajectory for DSL 2 A moving from 0.42 to 0.40 in 500 generations. The **orange** line is the trajectory for DSL 1 moving from 0.088 to 0.05

The population of Step 1 was *evolved forward in time*, following a demographic model. In particular, the founding population - considered to be isolated and homogeneous -, was expanded linearly to a larger population, by adding the same number of individuals, at each generation during the evolutionary expansion process. The targeted final population size was fixed to 10,000 indviduals (∼ 500 generations). In general, depending on the algorithm settings of the evolution-expansion process, all SNP markers are potentially allowed to be mutated according to a symmetric bi-allelic mutation model with a specific mutation rate (e.g. ∼ 10^−8^ per base pair per generation). Here, the mutation rate was defaulted to zero to make sure that all alleles stayed bi-allelic. At each generation, parents are chosen at random (random mating) and pass their genotypes to offspring according to Mendelian laws. Parental chromosomes can be allowed to recombine according to the fine-scale genetic map estimated from the data. Here, such recombinations were not allowed in order to preserve the initial linkage disequilibrium patterns of interest, by setting the Haldane genetic distances between SNPs to zero. Given that mating was completely at random, the last generation of the expanded population can thus be considered in Hardy-Weinberg equilibrium. Furthermore, the process uses a trajectory simulation method to control the frequency of the disease predisposing alleles (DPAs) of the DSLs: 0.05 for DSL 1 and 0.40 for DSL 2. The simulation starts from the pre-specified frequency of each DPA in the initial population and is restarted if the allele frequency at the present generation falls out of the desired range. The simulated trajectory forward in time over 500 generations is displayed in Fig. 3: The 2 loci DSL 1 and DSL 2 A (DSL 2B, DSL 2C, DSL 2D – see next section) were chosen as functional SNPs and buried in the LD block configuration referred to as setting A (B, C, D).

#### Step 3

Case-control samples were drawn according to a *rejection-sampling algorithm*. Samples were drawn following case/disease probabilities conditional on multi-locus genotypes, reflecting a target epistasis disease model (see Table 2 and the next Section).

#### Step 4

Output files were reformatted for subsequent analyses. In particular, the datasets obtained required appropriate reformatting in .PED and .MAP file formats, for visualization purposes of LD patterns with Haploview [34] and analyses with PLINK [23, 24] and MB-MDR [35].

Repetitive use of Steps 1-4 led to 400 (4 LD blocks × 100 replicates for a given effect size) datasets with 1000 case and 1000 control subjects each. These synthetic datasets had on average the same pre-specified disease predisposing allele frequencies in the expanded population (0.05 for DSL 1 and 0.40 for DSL 2) and followed the same disease known epistasis model as explained in the next Section.

### Generating a genetic disease trait determined by two epistatic causal loci DSL 1 and DSL 2

In what follows, we describe in more details the epistasis disease models implemented in this study. We considered four scenarios of DSL pairs: (DSL 1, DSL 2A), (DSL 1, DSL 2B), (DSL 1, DSL 2C), (DSL 1, DSL 2D).

The allele frequencies of the four selected DSLs in the initial founder population of 91 unrelated individuals and at the final expanded population of 10,000 individuals are tabulated in Table 1. We fixed the last generation allele frequencies, in line with observations from real-life data on Ankylosing Spondylitis, and to obtain the desired disease prevalence in the final population via the penetrance table (e.g., Table 2). This penetrance table represents one particular genetic epistasis model according to which case-control samples were drawn in a later stage.

**Table 1:** Allele frequencies of DSLs in founder and expanded populations (first allele in each pair is the minor allele)

		Minor allele frequencies *p*	
		Founder population	Expanded population
		91 individuals	10000 individuals
Causal SNP	Alleles		
DSL 1 (rs17644404)	A/T	0.09	0.05
DSL 2 A (rs10956767)	C/A	0.42	0.40
DSL 2 B (rs2073640)	T/C	0.33	0.40
DSL 2 C (rs1476427)	T/C	0.35	0.40
DSL 2 D (rs112698197)	T/C	0.19	0.40

**Table 2:** Imposed genotype penetrance table and disease prevalence calculation in the general population with allele frequencies under assumption of Hardy-Weinberg equilibrium

Genotype		Penetrance of genotype			Marginal
		−−−−−−−−−−−−−−−−			penetrance
		*AA*	*Aa*	*aa*	
		(1−*p*)^2^	2*p*(1−*p*)	*p* ^2^	
*BB*	(1−*p*)^2^	*p*(*D*|*G*_1_)	*p*(*D*|*G*_2_)	*p*(*D*|*G*_3_)	*M*_*x*_(*x*=1)
*Bb*	2*p*(1−*p*)	*p*(*D*|*G*_4_)	*p*(*D*|*G*_5_)	*p*(*D*|*G*_6_)	*M*_*x*_(*x*=2)
*bb*	*p* ^2^	*p*(*D*|*G*_7_)	*p*(*D*|*G*_8_)	*p*(*D*|*G*_9_)	*M*_*x*_(*x*=3)
Marginal		*M*_*y*_(*y*=1)	*M*_*y*_(*y*=2)	*M*_*y*_(*y*=3)	*p*(*D*)=*K*
penetrance					
	DSL 1	*AA=TT*	*Aa=TA*	*aa=AA*	
DSL 2 A		0.9025	0.095	0.0025	
*BB=AA*	0.36	0.0067	0.0911	0.0911	0.015
*Bb=CA*	0.48	0.0067	0.0392	0.0392	0.010
*bb=CC*	0.16	0.0067	0.0163	0.0163	0.008
Marginal		0.0067	0.054	0.054	p(D)=0.0113
penetrance					
		Odds ratio as compared to double homozygous *CC*/*TT* as baseline			
		*AA=TT*	*Aa=TA*	*aa=AA*	
*BB=AA*		1.00	14.88	14.88	
*Bb=CA*		1.00	6.05	6.05	
*bb=CC*		1.00	2.46	2.46	

In all settings, the minor allele frequency for DSL 1 is *p*=0.05 and for DSL 2 is *p*=0.40. Upper part: probabilities of disease given the genotype, values for simulated datasets in setting A (DSL 1 and DSL 2 A) with epistasis effect size *β*_3_=0.90 (see text). Lower part : odds ratio with major homozygous (TT) as baseline in setting A with epistasis effect size *β*_3_=0.90. The prevalence in the general population with this setting is around 1%

We considered four genomics configurations (A, B, C and D) of causal SNP pairs, fixing one locus (DSL 1) and allowing the second locus (DSL 2) involved in the causal SNP × SNP interaction to take different positions in an LD block. In setting A, both loci belong to a common LD block on chromosome 8 and are 56 kbp apart. In setting B, the second locus (DSL 2 B) is in a different LD block and 90 kbp separated from DSL 1, yet positioned in the middle of the LD block. In setting C, the second locus (DSL 2 C) is still in another LD block, 132 kbp further apart from DSL 1, this time residing at an edge of the LD block. Finally, in setting D, both loci are on different chromosomes: DSL 1 on chromosome 8 and DSL 2 D on chromosome 7. The positions of the four settings in their LD patterns are displayed on Figs. 1 and 2.

In Ankylosing Spondylitis, HLA-B*27 (playing the role of DSL 1 = rs17644404 in our synthetic datasets) was shown to be epistatic recessive on ERAP1 (playing the role of DSL 2 = rs10956767 in our synthetic datasets): the alleles of locus DSL 2 are masked when DSL 1 is homozygous (recessive) for the major allele T, or the alleles of locus DSL 2 only express themselves when epistatic locus DSL 1 has the dominant minor allele A [33, 36]. Both DSLs are suspected to be bi-allelic causal for Ankylosing Spondylitis. It has been shown that increased major allele dosage of DSL 2 is protective in HLA-B*27 positive subjects (i.e. at least one A allele) [33, 36]. The odds ratio for being affected is 2.5−3 times lower for homozygous major allele subjects on DSL 2 (ERAP1) than for homozygous minor allele on DSL 2 but only for HLA-B*27 positive subjects. These real-life results were taken as context to generate epistasis signals from a logistic regression model with varying effect size degrees of epistasis between DSL 1 and DSL 2. In particular, let *Y* be the binary outcome indicating the disease status (affected or unaffected) of an individual drawn from the current generation of the expanded population. This outcome is a Bernoulli random variable and if *π* denotes the probability for an individual to be affected, the model writes as:

1 $$\begin{array}{@{}rcl@{}} Y & \sim & \text{Bernoulli}(\pi) \end{array} $$

2 $$\begin{array}{@{}rcl@{}} \pi & = & \text{Pr}(Y=1 \, | \, g_{1}, g_{2}) \end{array} $$

3 $$\begin{array}{@{}rcl@{}} logit(\pi) & = & \beta_{0} + \beta_{1}\,\cdot\,g_{1} + \beta_{2}\,\cdot \, g_{2} + \beta_{3}\,\cdot\,g_{1}\cdot\,g_{2}  \end{array} $$

Here, the *β*_3_ term accounts for departure from additive main effects and measures the intensity of the interaction term, or of statistical epistasis beyond main effects (represented by *g*_1_ and *g*_2_). As we were not interested in joint two-locus effects but pure epistasis, we set *β*_1_=0, *β*_2_=0. In our simulation study, the real-life causal SNP pair reported in [33] was taken to be (DSL 1, DSL 2 A). Furthermore, in line with Evans and colleagues, a multiplicative effect on the odds ratio of affection status for the minor allele A of DSL 2 A, compared to the baseline was imposed (DSL 1/DSL 2 A = TT/CC). Each increase in A allele dosage of DSL 2 A multiplies the odds of affection status by a factor exp(*β*_3_)=1.65, 2.12, 2.46 in cases where *β*_3_ are 0.50, 0.75, 0.90 respectively, if and only if, there is at least one copy of allele A on DSL 1 locus. To meet this condition, the variables *g*1 and *g*2 of Eq. (3) were defined as follows:

$$\begin{array}{@{}rcl@{}} g1 & = & \left\{ \begin{array}{cl} 1 & \texttt{if DSL 2 A} = \texttt{(CC)} \\ 2 & \texttt{if DSL 2 A} = \texttt{(CA)} \\ 3 & \texttt{if DSL 2 A} = \texttt{(AA)} \end{array}\right.\\ g2 & = & \left\{ \begin{array}{cc} 0 & \texttt{if DSL 1} = \texttt{(TT)} \\ 1 & \texttt{otherwise} \end{array}\right. \end{array} $$

**Fig. 4:**
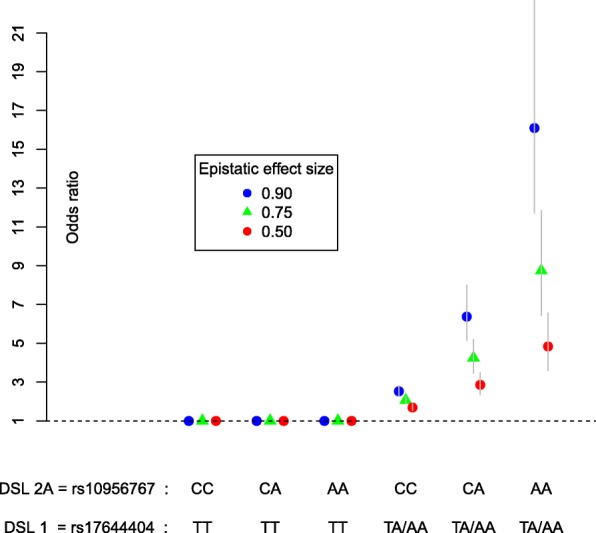
Disease odds ratios conditioned on the genotype of 2 causal loci: Odds ratio effect sizes conditioned on pure epistatic pairs of loci for disease status in the simulated case-control datasets. Causal effects for DSL 1 and DSL 2 A are conditioned on allele A for DSL 1. The risk allele A of DSL 2 A only increases risk for individuals carrying at least one copy of the DSL 1 risk allele (DSL 1 is epistatic to DSL 2 A). The low risk CC/TT genotype is set as the baseline (*OR*=1). The other genotype combinations are coded according to *g*1,*g*2 and their product *g*1×*g*2. Odds ratio are obtained by exponentiating the *β*_3_ coefficient of the interaction term from the logistic regression (see text). Error bars: 95% confidence intervals of possible odds ratio that are obtained in different simulated case-control samples

Achieving a disease prevalence similar to the estimated prevalence for Ankylosing Spondylitis of *p*(*D*)=*K*=0.5*%*−1.0*%*(=0.005−0.010), the parameter value for *β*_0_ was constrained to *β*_0_=−5. This defines all models parameters in Eq. (3). From these, penetrance values were obtained. For instance, for the epistatic pair (DSL 1, DSL 2 A) and *β*_3_=0.90, this resulted in Table 2. The corresponding odds ratios for each 2-locus genotype combination versus the reference CC/TT are depicted in Fig. 4. In total, four penetrance tables (times three effect sizes *β*_3_∈[0.50, 0.75, 0.90]), similar to Table 2, were built corresponding to the four LD position configurations described before (Figs. 1 and 2).

### Carrying out statistical epistasis analyses

Epistasis analyses on SNPs can be performed in an exhaustive fashion using all quality controlled data or on a reduced set of genetic markers. Both strategies can be motivated with several arguments but may lead to highly differing (even non-overlapping) results [37]. Most commonly, the set of input SNPs is reduced to remove high levels of correlation between markers that may lead to computational instabilities during epistasis modelling or to redundant epistasis. As this work focuses on the impact of LD on final epistasis results, we took the concept of LD as the basis for SNP-set reduction. The objective is to keep enough SNPs to maximize the chances that the discovery SNP-pair tags, or is in high correlation with, the actual causal SNP pair, but that unwanted between-SNP dependencies are minimized. In particular, for our simulated data, we implemented an LD pruning scheme that:

1. computed LD between SNPs within a sliding window of size 10 (i.e., 10 consecutive SNPs),
2. arbitrarily removed one element of the pair if the corresponding *r*^2^ was above a fixed threshold,
3. shifted the window with 2 SNPs forward and
4. repeated 1)-3) until all SNPs had been covered.

Practical applications to various datasets had pointed towards an informal *r*^2^ threshold of 0.75 [20]. Here, we formally investigated the *r*^2^=0.20 (“low” correlation), *r*^2^=0.50,*r*^2^=0.60,*r*^2^=0.75 as compared to no pruning at all as well.

As analytic tool we focused on Model-Based Multifactor Dimensionality Reduction (MB-MDR), in particular MB-MDR 4.4.1, which is written in C++ and runs stand alone from a command line or via scripting in UNIX/Linux environment [26, 38]. The core idea of MB-MDR is to pool 2-locus genotypes together which exhibit substantial statistical evidence towards increased or decreased disease risk, in which case, the multilocus genotype is labelled as “H” or “L”, respectively. No correction for main effects was performed as the simulated data involved pure epistasis only. Furthermore, MB-MDR’s final test statistic involved a case/control contrast test comparing “H” and “L” labelled multiloci genotypes. The latter overrules the default testing strategy in MB-MDR and omits individuals/multilocus genotypes for which no statistical evidence towards increased/decreased disease could be derived (i.e., omitting individuals/genotypes with the MB-MDR label “0” – see for more details for instance in [37]). This choice was motivated by three arguments:

1. the incorporation of “0” genotypes may blur the picture especially in synthetically controlled data [37];
2. contrast tests may be more powerful when there is a good balance between sufficient sample size and manageable multiple testing;
3. the comparative strategy MDR – to our knowledge the only multifactor dimensionality reduction method that has investigated the impact of LD on epistasis – forces all multiloci genotypes to be labelled as either “H” or “L” [39].

Finally, significance assessment was based on 999 permutations and the top 5000 SNP pairs (lowest *p*-values) were retained for performance assessment.

### Criteria to assess performance

The impact of LD pruning on binary classification as resulting from MB-MDR was measured via estimating power (i.e. statistical sensitivity) or estimating the probability of detecting the signals artificially introduced in the data. In particular, we used two operational definitions of sensitivity: Exact sensitivity: estimated as the number of times out of 100 (i.e., number of simulated datasets out of 100) where the true causal pair of SNPs is detected significant with MB-MDR’s multiple testing corrected *p*-value ≤ 0.05. Signal sensitivity: estimated as the number of times out of 100 (i.e., number of simulated datasets out of 100) where any of the SNP pairs tagging the causal pair is detected significant with MB-MDR’s multiple testing corrected *p*-value ≤ 0.05.

The second definition of sensitivity, i.e. signal sensitivity, requires knowledge about blocks of tag-SNPs around the causal SNPs and thus a threshold of allelic association. Here, we assumed two such thresholds: *r*^2^=0.20 and 0.45. To build the tag-SNPs list for each of the two causal locus at each threshold, we subset SNPs in *r*^2^≥0.20 or ≥0.45 with each of the causal locus (no window size restriction) from the complete SNPs set. The effect these thresholds has on the number of tag-SNPs of the causal SNP pairs (DSL 1, DSL 2 A), (DSL 1, DSL 2 B), (DSL 1, DSL 2 C) and (DSL 1, DSL 2 D) is tabulated in Table 4. Exact sensitivity was then estimated as the number of times out of the 100 simulated datasets where the true causal pair of SNPs was detected MB-MDR significant at a multiple testing adjusted *p*-value ≤ 0.05. Signal sensitivity was estimated as the number of times out of the 100 simulated datasets where any pair of tag-SNPs to the functional epistasis pair was detected MB-MDR significant at an adjusted *p*-value ≤ 0.05.

### Type I errors assessment

To explore the type I errors we created null data for which a complete randomization of the affection status was carried out across cases and controls i.e., no genetic association - main nor interaction with the trait. In these null data, correlations between all SNPs are kept fixed across replicates. Type I error was estimated as the proportion of 100 datasets for which at least one SNP pair (any SNP pair) was identified as significantly associated to the trait (MB-MDR with default options; thresholds for LD pruning LD(r2) < 0:75;LD(r2) < 0:60;LD(r2) < 0:50;LD(r2) < 0:20 and unpruned). Note that the occurrence of a significant SNP pair induces a significant block pair, and vice versa, irrespective of the block definition used. Variation is to be expected according the adopted pruning scheme.

## Results: LD impact on power

The simulation data consisted of 1200 synthetic datasets, corresponding to 4 LD blocks × 3 effect sizes × 100 retrospective case-control datasets with 1000 subjects in each cohort. The estimated heritabilities *h*^2^ are given in Table 3 and are all below *h*^2^=0.10. These were computed according to the subsequent formula (4), in which *G*_*i*_ represents the nine two-locus genotype combinations underlying *g*_1_×*g*_2_, and results immediately from the penetrance tables previously computed for each effect size (as Table 2 was an instance for *β*_3_=0.90 effect size and results in *h*^2^=0.083):

4 $$\begin{array}{@{}rcl@{}} h^{2} & = & \frac{{\sum\nolimits}_{i}^{9}\, [p(Y=1|G_{i})\cdot p(G_{i}) - p(Y=1)]^{2}}{p(Y=1)\,\cdot\, (1-p(Y=1))}  \end{array} $$

**Table 3:** Heritabilities associated to effect sizes for the epistatic interaction in all simulated datasets

Simulated	Interaction	Heritability
setting	*β*_3_	*h* ^2^
Effect size 1	*β*_3_=0.90	*h*^2^=0.083
Effect size 2	*β*_3_=0.75	*h*^2^=0.071
Effect size 3	*β*_3_=0.50	*h*^2^=0.059

**Table 4:** Tag SNPs number associated to causal variants for different LD(r2) values

Causal	Number of tag SNP at LD(*r*^2^) value:				
SNP	*r*^2^=0.20	*r*^2^=0.45	*r*^2^=0.55	*r*^2^=0.65	*r*^2^=0.75
DSL 1	60	2	2	1	1
DSL 2 A	115	114	114	111	98
DSL 2 B	110	110	109	107	107
DSL 2 C	81	80	80	78	78
DSL 2 D	76	48	31	31	24

Furthermore, Table 4 shows that only 1 SNP is in moderate to strong LD with the causal locus DSL 1 (*r*^2^ threshold of 0.75), while 60 SNPs are in very low LD with DSL 1 (*r*^2^ threshold of 0.20). Moderate to strong LD with DSL 2 A, B, C and D is observed for 98, 107, 78 and 24 SNPs (at *r*^2^ of 0.75), respectively. The number of tag SNPs (and thus the signal capture probability) increase with decreasing *r*^2^ threshold. For instance, for a threshold of 0.45, respectively 2, 114, 110, 80 and 48 tag-SNPs for DSL 1, DSL 2 A, B, C and D are obtained.

**Fig. 5:**
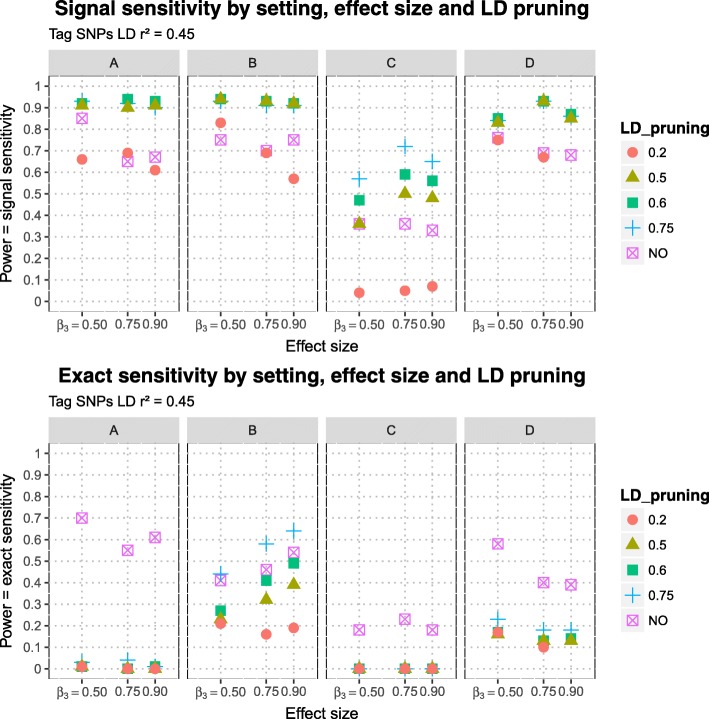
Sensitivities of MB-MDR to detect two-loci pure epistatic interaction in 4 settings at three effect sizes and with different LD pruning levels: Signal sensitivities (upper panel) and exact sensitivities (lower panel) are displayed at different LD pruning thresholds (unpruned data or LD pruning at 0.75, 0.60, 0.50 and 0.20). Signal sensitivities determined with tag-SNP subsets at LD *r*^2^≥0.45 with causal SNPs

**Fig. 6:**
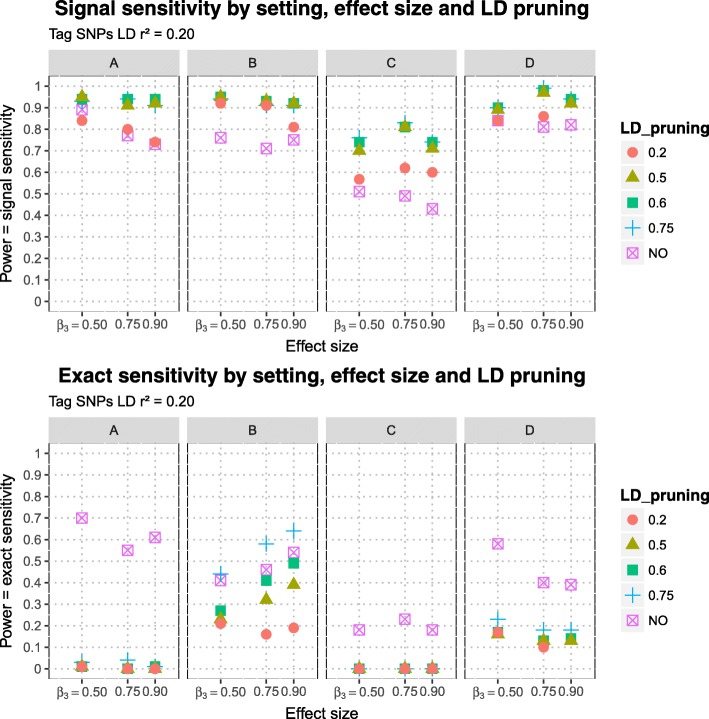
Sensitivities of MB-MDR to detect two-loci pure epistatic interaction in 4 settings at three effect sizes and with different LD pruning levels: Signal sensitivities (upper panel) and exact sensitivities (lower panel) are displayed at different LD pruning thresholds (unpruned data or LD pruning at 0.75, 0.60, 0.50 and 0.20). Signal sensitivities determined with tag-SNP subsets at LD *r*^2^≥0.20 with causal SNPs

The estimated signal sensitivities of MB-MDR to detect the simulated purely epistatic interaction (DSL 1, DSL 2), for different scenarios of DLS 2 position (DSL 2 A, DSL 2 B, DSL 2 C, DSL 2 D), three epistasis effect sizes and five LD pruning schemes before MB-MDR analysis are presented in Fig. 5, for signal sensitivity defined via *r*^2^≥0.45-tagging and in Fig. 6 for tagging determined by *r*^2^≥0.20. The estimated exact sensitivities are displayed on the lower panels of the aforementioned Figures. Note that estimates of exact sensitivity do not depend on block definitions. All estimates are tabulated in Table 5. The following observations are made:

1. For all scenarios of epistasis effect size and location of DSL 2, as well as tag-SNP block definition and pruning at different *r*^2^ values ranging from 0.20 to 0.75, the signal sensitivity is always higher than the exact sensitivity.
2. Also when no pruning is performed (thus all SNP pairs are screened for epistasis, regardless of between-SNPs correlations), the exact sensitivity is smaller than the signal sensitivity.
3. Exact sensitivities dramatically decrease when pruning is applied. The worst results are obtained for scenarios A and C, for which the corresponding DSL 2 can be considered to reside at the boundary of an LD (sub-)block. The results are only slightly better for scenario D. In case both DSLs are located on different chromosomes, exact sensitivity estimates range from 0.10-0.18 (setting D, see Fig. 1). In contrast, exact sensitivity estimates in case DSL 2 is located in the middle of an LD block range from 0.16-0.64, again depending on the epistatic effect size and LD pruning threshold (setting B, see Fig. 2).
4. Signal sensitivity can be further improved by SNP-set reduction via pruning. In general, the more LD-pruning is involved, the higher the signal sensitivity. Whatever the SNP-tag block definition used, too heavy pruning at *r*^2^ of 0.20 gives by far the lowest signal sensitivity. For all considered DSL 2 locations, little power (signal sensitivity) is lost by pruning further down from 0.75 to 0.60, retaining more SNPs. For setting C, power balances around 0.50 when more extensive pruning is done at *r*^2^ of 0.50 instead of 0.60, which is similar to flipping a coin and highly unacceptable (see Fig. 5).
5. There are no clear patterns regarding increasing epistasis effect size leading to increased exact or signal sensitivity.

**Table 5:** Sensitivity results of MB-MDR to detect two locus model of pure epistatic interaction in 1200 simulated datasets with real human genome LD patterns, for 3 effect sizes and after 5 LD pruning levels

LD block setting	LD pruning	Effect Size	Exact	Signal Sensitivity	
			Sensitivity	−−−−−−−−−−−−−−−−−−	
				*tag-SNP condition*	*tag-SNP condition*
				LD *r*^2^≥0.45	LD *r*^2^≥0.20
*A*	unpruned	*β*_3_=0.90	0.61	0.67	0.73
Two SNPs		*β*_3_=0.75	0.55	0.65	0.77
in same		*β*_3_=0.50	0.70	0.85	0.89
LD block	LD *r*^2^≤0.75	*β*_3_=0.90	0.01	0.90	0.91
		*β*_3_=0.75	0.04	0.92	0.94
		*β*_3_=0.50	0.03	0.93	0.93
	LD *r*^2^≤0.60	*β*_3_=0.90	0.01	0.93	0.94
		*β*_3_=0.75	0.00	0.94	0.94
		*β*_3_=0.50	0.01	0.92	0.94
	LD *r*^2^≤0.50	*β*_3_=0.90	0.00	0.91	0.92
		*β*_3_=0.75	0.00	0.90	0.91
		*β*_3_=0.50	0.01	0.91	0.95
	LD *r*^2^≤0.20	*β*_3_=0.90	0.00	0.61	0.74
		*β*_3_=0.75	0.00	0.69	0.80
		*β*_3_=0.50	0.01	0.66	0.84
*B*	unpruned	*β*_3_=0.90	0.54	0.75	0.75
Two SNPs		*β*_3_=0.75	0.46	0.70	0.71
in middle		*β*_3_=0.50	0.41	0.75	0.76
of two	LD *r*^2^≤0.75	*β*_3_=0.90	0.64	0.91	0.91
separate		*β*_3_=0.75	0.58	0.91	0.91
LD blocks		*β*_3_=0.50	0.44	0.93	0.94
	LD *r*^2^≤0.60	*β*_3_=0.90	0.49	0.92	0.92
		*β*_3_=0.75	0.41	0.93	0.93
		*β*_3_=0.50	0.27	0.94	0.95
	LD *r*^2^≤0.50	*β*_3_=0.90	0.39	0.92	0.92
		*β*_3_=0.75	0.32	0.93	0.93
		*β*_3_=0.50	0.23	0.94	0.95
	LD *r*^2^≤0.20	*β*_3_=0.90	0.19	0.57	0.81
		*β*_3_=0.75	0.16	0.69	0.91
		*β*_3_=0.50	0.21	0.83	0.92
*C*	unpruned	*β*_3_=0.90	0.18	0.33	0.43
One SNP		*β*_3_=0.75	0.23	0.36	0.49
in a block		*β*_3_=0.50	0.18	0.36	0.51
and one	LD *r*^2^≤0.75	*β*_3_=0.90	0.0	0.65	0.74
in the edge		*β*_3_=0.75	0.0	0.72	0.83
of a separate		*β*_3_=0.50	0.0	0.57	0.76
LD block	LD *r*^2^≤0.60	*β*_3_=0.90	0.0	0.56	0.74
		*β*_3_=0.75	0.0	0.59	0.81
		*β*_3_=0.50	0.0	0.47	0.74
	LD *r*^2^≤0.50	*β*_3_=0.90	0.0	0.48	0.71
		*β*_3_=0.75	0.0	0.50	0.81
		*β*_3_=0.50	0.0	0.36	0.70
	LD *r*^2^≤0.20	*β*_3_=0.90	0.0	0.07	0.60
		*β*_3_=0.75	0.0	0.05	0.62
		*β*_3_=0.50	0.0	0.04	0.57
*D*	unpruned	*β*_3_=0.90	0.39	0.68	0.82
Two SNPs		*β*_3_=0.75	0.40	0.69	0.81
on LD blocks		*β*_3_=0.50	0.58	0.76	0.84
of separate	LD *r*^2^≤0.75	*β*_3_=0.90	0.18	0.86	0.94
chromosomes		*β*_3_=0.75	0.18	0.93	0.99
		*β*_3_=0.50	0.23	0.84	0.90
	LD *r*^2^≤0.60	*β*_3_=0.90	0.14	0.87	0.94
		*β*_3_=0.75	0.13	0.93	0.98
		*β*_3_=0.50	0.17	0.85	0.90
	LD *r*^2^≤0.50	*β*_3_=0.90	0.13	0.85	0.92
		*β*_3_=0.75	0.13	0.93	0.97
		*β*_3_=0.50	0.16	0.83	0.89
	LD *r*^2^≤0.20	*β*_3_=0.90	NA	NA	NA
		*β*_3_=0.75	0.10	0.67	0.86
		*β*_3_=0.50	0.17	0.75	0.84

## Results: LD impact on type I error

LD between SNPs gave rise to conservative performance of MB-MDR. Type I error estimates were below 1% for all LD block scenarios and every LD pruning thresholding (see Table 6).

**Table 6:** False positive rates (type I error) estimation in % for different LD patterns and pruning levels

LD	LD block settings:			
pruning	*A*	*B*	*C*	*D*
unpruned	< 1%	< 1%	< 1%	< 1%
LD (*r*^2^) 0:75	< 1%	< 1%	< 1%	< 1%
LD (*r*^2^) 0:60	< 1%	< 1%	< 1%	< 1%
LD (*r*^2^) 0:50	< 1%	< 1%	< 1%	< 1%
LD (*r*^2^) 0:20	< 1%	< 1%	< 1%	< 1%

Null data with no disease association to the investigated pair of SNPs as disease susceptibility loci

The type I error estimates from the null data suggest that a two-locus test between two SNPs does not occur frequently by chance, whatever the LD blocks settings. The fact that no signals were identified in the null data may be somewhat surprising. In Cattaert et al. [37], type I error estimates were around 5% for all scenarios considered, in line with the property of step-down maxT p-value adjustments, in that at least weak control of FWER is ensured. Notably, their simulated null data assumed no LD between markers. Here, strong LD between markers may induce violations of the maxT’s subset pivotality assumption [40]. It seems that for the genotype data we generated, based on real-life LD patterns, the epistasis detection procedure is over conservative. Whether this holds in general for null data with correlated SNPs, warrants further investigation. On the positive side, these results do not downplay previously obtained power estimates.

## Discussion

The detection of biological epistasis via SNPs remains one of the biggest challenges in genetic epidemiology due the inherent computational, mathematical/statistical complexities of the problem. Some of these complexities include the curse of dimensionality, the winner’s curse, genetic heterogeneity, absence of main effects, redundancies or dependencies (LD) between SNPs. The present study investigates the effect of real human linkage disequilibrium patterns on gene-gene interaction detections. The LD patterns were extracted from the Human 1000 Genomes Project public repository database ([30], see also International HapMap Consortium [28, 29]). Different scenarios of pure epistatic effect sizes in different LD block combinations for a non-fully penetrant genetic model of a disease of interest were simulated. The minor allele frequencies of the causal variants were set to 0.05 and 0.40 so as to mimick Ankylosing Spondylitis disease prevalence in a general population [33]. The broad sense heritabilities associated to the pure epistatic interaction effect sizes in our simulated datasets were *h*^2^ = 0.059, 0.071, 0.083, simulating a narrower range in effect sizes than in the Grady et.al. pioneering study [39]. The genetic model implemented in our study along with the odds ratios effect sizes for disease risk and the genotype penetrance table were inspired from the suspected pure biological epistatic effect between ERAP1 and HLA-B*27 in Ankylosing Spondylitis affecting the general population with British ancestry with a prevalence of ∼ 1*%* [33, 36].

### Realistic simulations to investigate the impact of GPD on epistasis analyses

We are not the first ones who studied LD in the context of epistasis screening. In relation to multifactor dimensionality reduction strategies, we are aware of the work of Grady and co-authors [39]. Our study differs from theirs in several ways:

- We considered LD patterns from real-life data, in particular from the HapMap3 and 1000 Genomes Project, rather than customized LD profiles. Indeed, in their work, Grady et al. [39] did not use real LD patterns from HapMap projects; The HapMap3 and 1000 Genomes Project data were not available in 2011. They simulated their own LD patterns instead, using a software called genomeSIMLA that is no longer supported and fails to compile with current versions of C++ compilers. Other software packages producing real LD pattern, such as Hapgen2 [41], cannot directly implement epistatic interactions between genetic loci; appropriate R packages need to be used in complement to Hapgen2. Alternatively, epiSIM [42] to simulate epistasis with Markov Chains can be employed, but again independent from real-life LD patterns or pairwise SNP correlation structures. We have developed our own scripts in Python, using the simuPOP libraries [31, 32], to combine both real-life templated genomic data generation with epistasis models of interest superimposed.
- We took the LD patterns from a presumably homogeneous single subpopulation (GBR ancestry). We checked that this subpopulation could be considered unstructured using the genomic control algorithm approach [43] and the fixation index approach (results not presented here). Our datasets did not show evidence of stratification. The presence of individuals from different populations with different genetic origins within a panel can produce LD between unlinked loci because of differences of allele frequencies. Such stratification can lead to a bias estimates of LD, which may increase the rate of false positive LD structure [44]. Notably, genotyped individuals in the sample that are not independent may also lead to biased estimations of LD [45]. Causal SNP pairs were selected in such a way that they covered different areas of an LD block (e.g., in the middle or at a boundary).
- We fully and explicitely defined pure epistasis for a causal SNP pair based on epistasis findings in Ankylosing Spondylitis [33, 36], one of the rare evidences for replicable epistasis with a biological underpinning in humans.
- We used relatively small epistasis effect sizes resulting in a narrow range of heritabilities. Polderman et al. [46] observed that for most human complex traits, out of nearly 18,000 traits, the trait variation can mainly be attributed to additive genetic variation. Hence, we feel that our heritability range *h*^2^<10*%* may be more realistic than the ones investigated by Grady et al. [39], in the range of 5*%*−25*%*.
- We assessed the impact of LD pruning, before, and exploitation of various tag-SNP block definitions, after, the analysis with the (MB)-MDR algorithm on statistical sensitivity, using *r*^2^ as a measure of LD. Grady and co-authors were one of the first authors to consider signal sensitivity and the impact of LD in the context of epistasis screening and dimensionality reduction. They defined signal sensitivity by the number of times out of 100 synthetic data replicates for which MDR chose a best model involving SNPs for which the measure of association between SNP and functional locus was *D*^′^≥0.90. We used *r*^2^ as it can be used as both a measure of LD and GPD, and it is commonly used in the context of genome-wide association studies with a direct interpretation. Indeed, a GWAS sample size must be increased by a factor of 1/*r*^2^ to detect an unmeasured genetic variant, compared to the sample size for testing the variant itself. Analytically, a vast number of tools exist to identify statistical epistasis using SNPs (see for overviews and references for instance in [15, 21, 37, 47]). This number is likely to increase with investigators from deep learning communities entering the field. We singled out one such tool, namely Model-Based Multifactor Dimensionality Reduction (MB-MDR). At its conception in 2007-2008 [48, 49], it was templated on principles of Multifactor Dimensionality Reduction (MDR) [50]. It further developed into a framework dealing with some of the shortcomings of MDR, which are described and discussed in [37], dealing with different trait types (binary, continuous, time-to-survival, censored) and study designs (independent or related individuals). Compared to MDR and related multifactor dimensionality reduction methods [51], MB-MDR breaks with cross-validation testing but dedicates computation time to appropriate association (contrasts) tests for the data at hand and a resampling-based Westfall and Young step-down maxT adjusted *p*-values implementation [40] to assess statistical significance of SNP pairs [26]. The MB (Model-Based) part of MB-MDR mainly refers to the ability to adjust for lower-order effects and to test for epistasis conditional on main effects [52].

### Flexible definitions of sensitivity and false positive rate

It is no surprise that also in our study signal sensitivity estimates exceed exact sensitivity (results observations 1 and 2), as for the first, the signal is expanded over sets of genetic markers comprised of at least 2 SNPs. It makes sense to define such sets based on SNPs being in LD, but alternative definitions are possible (see later). The larger the sets of proxy’s to the functional SNPs, the larger the capture probability of the disease signal. Over all considered simulation settings, the exact sensitivities were in the range of 18*%*−70*%* for unpruned data (compared to 93*%*−100*%*, in the absence of LD between simulated markers, as in [37]. This suggests that the presence of LD may not be as a merit in GWAIS as it is in main effects GWAS, even in relatively small datasets (i.e., number of markers) for which the multiple testing burden is less pronounced. Notably, any definition of power should be seen in the context of the test’s performance on type I error control. Depending on the method, type I error control may refer to different things. For instance, let us take the example of MB-MDR and MDR, both belonging to the same family of epistasis detection tools, namely those relying on an internal multifactor dimensionality reduction step of multiloci genotypes. In a detailed study of MB-MDR for binary traits [37], we have computed false positive rates for MB-MDR as the proportion of null data sets that highlight at least one significant MB-MDR pair (corrected for multiple testing). For MDR the equivalent is the proportion of null data sets for which the best model is found significant. Hence, this false positive rate is a simple rate for MDR which only proposes a single best model, but evaluates family-wise (FWER) for MB-MDR which possibly reveals multiple (competing) significant epistasis models. Regardless of these different connotations, we showed before that MB-MDR adequately maintains FWER to 5% in a variety of error-free scenarios, whereas MDR showed a tendency for slightly elevated FWER estimates. The considered error-free scenarios assumed no dependencies between genetic markers, though. It is to be expected that FWER increases with increasing degrees of allelic association between SNPs. Studying this in depth, hereby comparing multiple epistasis tools comprising representatives of the entire spectrum of machine learning, mathematical and statistical methodologies was beyond the scope of this study, but would be valuable, especially when extending the concept of FWER from exact to signal FWER in a similar way as we have done for “power” (exact, signal sensitivity).

The type I error estimates from the null data suggest that a two-locus test between two SNPs does not occur frequently by chance whatever the LD blocks settings.

The fact that no signals were identified in the null data may be somewhat surprising. In Cattaert et al. [37], type I error estimates were around 5% for all scenarios considered, in line with the property of step-down maxT *p*-value adjustments, in that at least weak control of FWER is ensured. Notably, their simulated null data assumed no LD between markers. Here, strong LD between markers may induce violations of the maxT’s subset pivotality assumption [51]. It seems that for the genotype data we generated, based on real-life LD patterns, the epistasis detection procedure is over conservative. Whether this holds in general for null data with correlated SNPs, warrants further investigation. On the positive side, these results do not downplay previously obtained power estimates.

A bit of a surprise was the absence of a clear relationship between epistasis effect size and power (results observation 5), for both exact and signal sensitivity assessments. On the other hand, the same observation was also made before by Grady et al. [39]. In addition, comparison of Fig. 5 with Fig. 6 suggests a complex interplay between tag-SNP block definition and pruning scheme. Our block definitions based on *r*^2^ of 0.20 or 0.45 may not make much sense on real-life data, but they do show that it is absolutely insensible to prune the data at lower thresholds than used to define tag-SNP blocks.

### Pre-analysis LD pruning thresholds

The drawback of LD-pruning SNPs to reduce the number of SNPs included in an epistasis screening is that it may eliminate true causal variants from the search pool and thus that technically, the only way to detect the epistasis signal is by using tag-SNP block definitions. We have seen that exact sensitivity is highly dependent on the LD-patterns in which the causal loci are hidden (results observation 3). In setting C (DSL 1 × DSL 2 C), DSL 2 C causal SNP is at the boundary of an LD block (see Fig. 2); this setting performed the worst in terms of exact sensitivity (notably, even in terms of signal sensitivity). However, there is also a clear added value of performing LD-pruning prior to an epistasis screening. LD pruning increases the signal sensitivity as compared to no pruning at all (results observation 4). A possible explanation is that LD pruning results in removal of multicollinear pairs of SNPs, which would otherwise lead to redundant epistasis top findings and would hamper other pairs of SNPs to reach statistical significance due to the multiple testing burden. Too liberal LD pruning (e.g. at *r*^2^≤0.20) will make the difference between causal SNP and tag-SNP block around it too little and thus signal sensitivity will converge to exact sensitivity. In our simulated data, for all settings (A, B, C and D) and for the signal sensitivity calculated with the largest tag-SNP subset (retaining SNPs with correlations *r*^2^≥0.20 to the causal SNPs), there was not much difference in signal sensitivity achieved after pruning, whether the LD pruning was done at *r*^2^= 0.75, 0.60 or 0.50. This is not surprising, given the data reported in Table 4: the number of tag-SNPs for DSL 2 A, B and C hardly varied when an *r*^2^ threshold of 0.20-0.75 was used. The situation is different for DSL 2 D, but also here the number of tag-SNPs was fairly stable for *r*^2^ thresholds of 0.55-0.75. Hence, although seemingly harsher *r*^2^ pruning may give similar performance, it may not have resulted in larger reductions in number of SNPs for MB-MDR testing. Therefore it is important to thoroughly understand the LD/GPD structure in the data and to adequately estimate SNP correlations, in unbiased ways.

### Post-analysis LD block algorithms

Our results raise questions about how LD-pruning algorithms or LD-block estimation algorithms actually work. For instance, what is the behaviour of these algorithms at boundaries of LD blocks? Are SNPs at the boundary of such a block more likely to be filtered out during LD-pruning? Is it sensible to work with asymmetric tag-SNP blocks, such as those induced by LD-blocks around SNPs? Regarding pruning strategies, earlier work of ours and unpublished work already showed their impact on final epistasis results, across different analytic epistasis detection tools [53]. For instance, whereas BOOST (logistic regression - [54]) generated over 2000 statistically significant interaction SNP pairs after pruning, a non-overlapping set of 200 significant SNP pairs were obtained when no pruning was applied prior to epistasis modelling. On the same unpruned SNP set of 500,000 SNPs MB-MDR (dimensionality reduction) generated 6500 statistically significant interaction SNP pairs, whereas a subset of approximately half of the aforementioned 6500 significant SNP pairs after pruning. Whereas MB-MDR’s maxT based significance assessment suffers from false positives, due to harmful multicollinearity between SNPs, more than BOOST’s Bonferroni correction, it is comforting that MB-MDR’s strategy for the detection of interacting SNP pairs does not increase the number of significant results when reducing the input SNP-set, in contrast to BOOST’s most commonly used implementation. Hence, when interpreting epistasis findings one cannot decouple the data preparation step (incl. reduction of SNPs, possibly based on prior knowledge about biological interactions – see also Biofilter [55]) from the characterizing components of the chosen analytic tool and the implemented significance assessment algorithms (incl., multiple testing correction), as also argued in [21]. It would be interesting to see new machine learning based epistasis detection tools with built-in “minimum redundancy maximum relevance” feature selection procedures. In general, a feature selection (pruning) scheme is of interest that chooses (resp., results in) a subset of SNPs that can predict others with small probability error. Notably in [53], pruning was performed considering sliding windows of size 50 (i.e., 50 SNPs) with window increments of 1 marker. For any pair of markers in such a window with *r*^2^>0.75 the first SNP in the pair was discarded, as implemented in SVS Version 7.5 (Golden Helix, Inc.) [56]. PLINK 1.07/1.9 take a different approach than SVS: SNPs are recursively being removed in sliding windows based on a Variance Inflation Factor (VIF) threshold to detect multicollinearity between SNPs or on pairwise SNP correlations *r*^2^ with a greedy SNP removal procedure and *r*^2^ based on genotypic correlations or via maximum likelihood phasing. These pruning strategies assume that redundancy is removed within a window, which is different from an LD-block, defined as a set of consecutive genetic markers with relatively little recombination within. Other methods explicitly take into account reference genotypes to determine LD-blocks and to select tag-SNP representatives in bins of highly correlated SNPs (e.g., see references in [57]). Sliding window based rather than LD-block based tag-SNP selection is not so much of a problem when the aim is to remove multicollinearity between SNPs, but surely is when interpreting epistasis results towards causality and bridging the gap between statistical and biological epistasis.

There are several ways to detect LD-blocks. One is the four gamete test of Hudson and Kaplan (1985) [58]. These authors defined a segment of bi-allelic SNPs as a “block” if between every pair of SNPs at most 3 out of 4 gametes were observed. Another is based on rejecting the hypothesis that 95% of pairs of SNPs in the “block” are in linkage equilibrium. Yet another is based on “haplotype blocks”, representing regions that are inherited without substantial recombination in the ancestors of the current population [59]. Haplotype blocks truly rely on the concept of LD – allelic association and linkage – commonly being measured by Pearson correlation *r*^2^. In essence, the latter measure is only a measure of allelic association and restricting to sliding windows ignores the fact that LD-blocks vary in length depending on the occurrence of recombination sites. Several studies have been built around understanding and estimating recombination rates in human genomes (e.g., [60]). Since the beginning of the 21st century, with the availability of HapMap data, several empirical strategies have been proposed to detect the boundaries of haplotype blocks with reference panels (see [61] and references therein). However, depending on study design and analytic strategy for LD estimation, biased estimates may be obtained [62]. Several analyses using *r*^2^, such as in the PLINK software (1.07), hypothesize that the extent of *r*^2^ around the causal polymorphism depends only on a drift-recombination process in a randomly mating population without selection. In real life data, this assumption may be violated [63]. Also, in highly related samples, *r*^2^ overestimates the true LD value. This is of a concern when related samples are used for epistasis analysis, such as in FAM-MDR [64], and appropriate corrections need to be made for kinship when estimating LD-block structures (Mangin et al. [65]). The estimate proposed by Mangin and co-authors also corrects for population structure, which is useful in the context of multi-center meta-epistasis analyses and interpreting results at the level of tag-SNP blocks, in line with our simulation study. As the true functional SNP pairs are typically unknown in real-life data, more work is needed to define significant interaction at the block-level, similar to developments in epistasis research that take genes as units of analyses [66].

## Conclusions

There is a clear advantage of removing SNP redundancy prior to statistical epistasis screening in the search for gene × gene interactions with SNP panels: pruning avoids increases in false positives (redundant epistasis), due to multicollinear SNPs or due to multiple testing strategies that inadequately take dependencies between tests into account. Such dependencies may be highly complex and driven by complex LD patterns between SNPs, which are population dependent. This advantage comes with a caveat, namely that important actors may have been eliminated, hereby reducing exact sensitivity (i.e., the power to detect the exact functional interacting pair of SNPs). Although we have exemplified this for the first time in the context of Model-Based Multifactor Dimensionality Reduction as analytic epistasis detection framework, we have argued that similar conclusions are to be expected in other contexts as well, although with different degrees of impact.

LD-pruning based on *r*^2^ at a threshold of 0.75, proposed by Gusareva et al. [20], remains to be an overall good strategy in the synthetic scenarios considered. It optimises signal sensitivity compared to no pruning at all. Specific data contexts may allow lowering this threshold; this is making the definition of redundancy between markers less stringent. There is a complex interplay between the adopted pruning strategy prior to epistasis screening and the adopted LD-block definition or assessment, that both determine the impact on signal sensitivity. This is already the case when restricting attention to a single pruning methodology (e.g., removal of SNPs via pairwise SNP correlations *r*^2^ exceeding a threshold within sliding windows), only varying the threshold for redundancy. At this pre-analysis stage, we are not concerned about the causes of multicollinearity, which could be a mere artefact of the collected samples without any biological or population evolutionary underpinning. We are concerned with such causes and unbiased estimation of LD-blocks for the interpretation of epistasis results at the LD-block level, rather than at the SNP level.

## Code availability

The MB-MDR software can be downloaded from http://bio3.giga.ulg.ac.be/. The algorithms to compute both exact sensitivity and signal sensitivity are implemented in a customized Python program embedded in a job script to scan 100 MB-MDR output files automatically, for all considered scenarios. Both programming code and simulated data are available upon request (marc.joiret@uliege.be).
