# Correction: Confounding of linkage disequilibrium patterns in large scale DNA based gene-gene interaction studies

**DOI:** 10.1186/s13040-022-00296-9

**Published:** 2022-04-11

**Authors:** Marc Joiret, Jestinah M. Mahachie John, Elena S. Gusareva, Kristel Van Steen

**Affiliations:** 1 BIO3, GIGA-R Medical Genomics, Avenue de l’Hôpital 1-B34-CHU, 4000 Liège, Belgium; 2 Biomechanics Research Unit, GIGA-R in-silico medicine, Liège, Avenue de l’Hôpital 1-B34-CHU, 4000 Liège, Belgium; 3 WELBIO researcher, Avenue de l’Hôpital 1-B34-CHU, 4000 Liège, Belgium

**Correction to: BioData Min 12, 11 (2019)**

10.1186/s13040-019-0199-7

Following publication of the original article [1], the authors identified an error in the heritability estimation. In particular, formula (4) should read as

$$\frac{\sum_{i=1}^{9}\,p(G_i)\cdot\big[p(Y=1|G_i)-p(Y=1)\big]^2}{p(Y=1)\,\cdot\,(1-p(Y=1))}$$

This has however no impact on the trustworthiness of the results; heritability estimates were merely provided as descriptive information.

Discussion elements remain unchanged.
